# Crosstalk between exosomes and autophagy in spinal cord injury: fresh positive target for therapeutic application

**DOI:** 10.1007/s00441-022-03699-6

**Published:** 2022-11-16

**Authors:** Rui-yu Li, Qi Hu, Xu Shi, Zhen-yu Luo, Dong-hua Shao

**Affiliations:** 1 Anqing First People’s Hospital of Anhui Medical University, Anqing, 246000 Anhui Province, China; 2 Jiangsu University, Zhenjiang, 212001 Jiangsu Province, China

**Keywords:** Exosomes, Autophagy, Autophagosome, Spinal cord injury, Extracellular vesicles, Stem cells

## Abstract

Spinal cord injury (SCI) is a very serious clinical traumatic illness with a very high disability rate. It not only causes serious functional disorders below the injured segment, but also causes unimaginable economic burden to social development. Exosomes are nano-sized cellular communication carriers that exist stably in almost all organisms and cell types. Because of their capacity to transport proteins, lipids, and nucleic acids, they affect various physiological and pathological functions of recipient cells and parental cells. Autophagy is a process that relies on the lysosomal pathway to degrade cytoplasmic proteins and organelles and involves a variety of pathophysiological processes. Exosomes and autophagy play critical roles in cellular homeostasis following spinal cord injury. Presently, the coordination mechanism of exosomes and autophagy has attracted much attention in the early efficacy of spinal cord injury. In this review, we discussed the interaction of autophagy and exosomes from the perspective of molecular mechanisms, which might provide novel insights for the early therapeutic application of spinal cord injury.

## Introduction

More than 1 million people worldwide are disabled due to SCI (Tahmasebi and Barati 2022). Due to the different extent and location of the injury, patients experience sensory and motor disturbances in different locations and further develop neuropathic pain (Cheng et al. 2021b). The pathophysiological mechanism of SCI is relatively complex and should be further explored. At present, the doctrine of a two-step process is widely accepted, namely, primary and secondary damage mechanisms. The mechanism of primary injury mainly involves the changes in the internal shape of spinal cord caused by severe external forces and the destruction of the normal internal physiological microenvironment (Nicaise et al. 2022). There is abundant evidence that the primary damage mechanism can lead to a cascade of secondary damages such as ischemia, demyelination, inflammation, apoptosis, and neuronal necrosis (Wang et al. 2021a). The mechanism of secondary damage is caused by a variety of cellular interactions, including macrophages, activated microglia, reactive astrocytes, oligodendrocyte precursor cells, etc. (Li et al. 2021b). In addition, some theories divide the pathophysiological process of SCI into four stages: acute phase (0 to 48 h), subacute phase (2 to 14 days), intermediate phase (2 weeks to 6 months), and chronic phase (over 6 months) (Ahuja and Fehlings 2016).

Repair of the damaged spinal cord remains a major clinical challenge. Several common methods of treating SCI work to promote neuroprotection, angiogenesis, immunomodulation, and axonal regeneration (Li et al. 2021c; Tran et al. 2022). Both exosomes and autophagy are candidates for treatment options after SCI, and there are numerous related reports, respectively. Exosomes may carry beneficial or harmful cargoes to participate in its pathological response process, resulting in varying degrees of alleviation or aggravation. Similarly, the process of autophagy also exists in the pathological response after SCI, which may alleviate the inflammatory response by degrading waste products or aggravate the pathological process by damaging normal neuronal cells. Interestingly, there is shared information between exosome biogenesis and autophagy, which may promote or antagonize each other.

## The biogenesis of exosomes

**Fig. 1:**
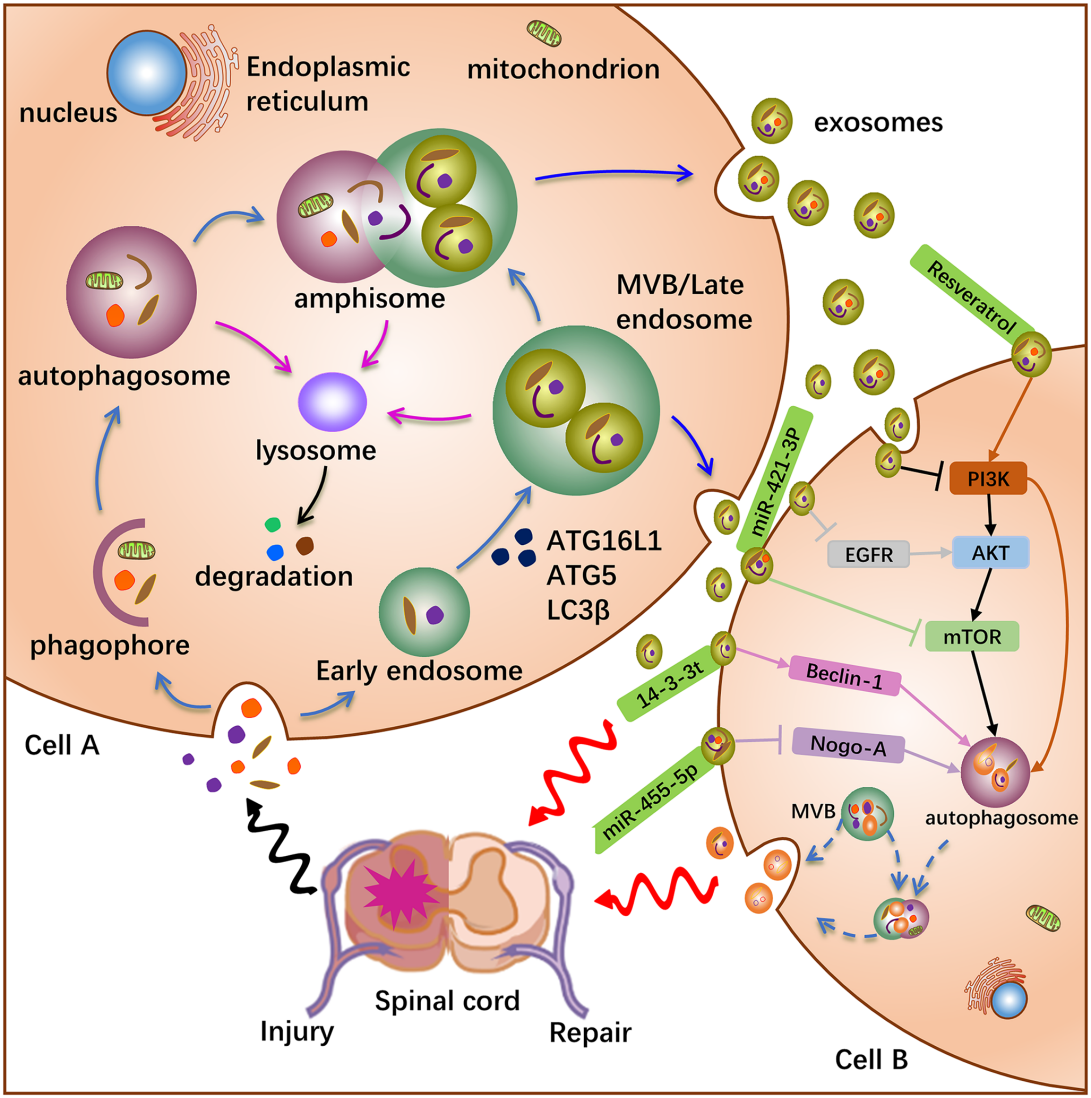
Crosstalk between exosomes and autophagy. Following SCI, the body releases signals to associated cells, stimulate them to produce early endosomes, ILVs then aggregate to form late endosomes or MVBs. The autophagosome is formed synchronously, and then MVBs fuse with it to produce the amphisome. Exosome secretion: ^①^ MVBs through fusion with the plasma membrane, ^②^ amphisome through fusion with the plasma membrane. Exosome biogenesis requires the participation of autophagy-related proteins ATG16L1, ATG5, and LC3β. In addition, exosomes containing informational substances increased the number of autophagosomes through molecular/signaling pathways, thereby activating autophagy. Additionally, lysosomes can degrade and recycle cargo carried by autophagosomes, amphisome, and MVBs. Exosomes containing cargo are transported to the injury site, which may reduce inflammation, inhibit apoptosis, and protect axons, and may also stimulate adjacent cells to secrete exosomes to repair the injury

According to the International Society for Extracellular Vesicles Guidelines in 2018, extracellular vesicles (EVs) are present in almost all human cells and transmit beneficial or harmful information from cell to cell (Théry et al. 2018). Exosomes (30–150 nm) are a subtype of EVs released by eukaryotic cells into the external space (Yáñez-Mó et al. 2015). EVs also include larger vesicles, such as macrovesicles (150–1000 nm) and apoptotic bodies (1–5 μm) (Tschuschke et al. 2020). In addition to the requirements for diameter, exosomes also have a series of molecular markers that are usually derived from endosomes, such as CD63, CD81, and CD9 (Kowal et al. 2016). As the carrier of proteins, RNA, and lipids delivery, exosomes are one of the important modes of intercellular communication. The biogenesis of exosomes requires two transport hubs: early endosomes and late endosomes (Scott et al. 2014). First, various substances from the outside are introduced into the cell by fusion with the plasma membrane to form early endosomes; then, it further encapsulates the nucleic acids, proteins, and lipids in the cell to form intraluminal vesicles (ILVs). The gathering of ILVs leads to the gradual transformation of early endosomes into multivesicular bodies (MVBs), which continue to fuse to form late endosomes (LEs). Finally, LEs fuse with the plasma membrane to secrete the vesicles into the extracellular space, forming exosomes. However, not all LEs fuse with the plasma membrane to secrete exosomes; some associate with lysosomes, leading to recycling or degradation of cytoplasmic components and organelles (Raposo and Stoorvogel 2013). Furthermore, LEs or MVBs fuse with double-membrane vesicles termed autophagosomes to form amphisome, which further fuse with the plasma membrane to secrete exosomes into the outer space (Hessvik and Llorente 2018) (Fig. 1). In addition, autophagosomes also associate with lysosomes to degrade carried cargo (Lamb et al. 2013).

The formation of exosomes involves a specific sorting mechanism, and the biogenesis of MVBs requires endosomal sorting complex required for transport (ESCRT) mechanism (Hurley 2015). ESCRT is a membrane-shaping protein that recognizes ubiquitination-modified membrane proteins and mediates endocytic vesicle budding and MVBs formation, thereby promoting exosome production (Vietri et al. 2020). ESCRT consists of four complexes (ESCRT-0, -I, -II, and -III) and associated proteins (vacuolar protein sorting-associated protein 4 (Vps4), tumor susceptibility 101 (Tsg101), and apoptosis-linked gene 2-interacting protein X (Alix)), which function in a specific order (Vietri et al. 2020). First, ESCRT-0 recognizes ubiquitin-tagged proteins; then ESCRT-I and -II cooperate to induce endocytic cargo of the early endosomes, which encapsulate the specifically tagged contents to form buds. The bud is subsequently dissociated from the early endosome under the action of ESCRT-III to form ILVs; at the same time, the ESCRT complex is dissociated from the endosomal membrane under the action of the ATPase Vps4, and continues to perform biological functions (Henne et al. 2013). Although ESCRT has long been required for ILVs formation, in ESCRT-depleted cells, ILVs were still observed within MVBs, suggesting an ESCRT-independent way of forming ILVs in cells. The research team found that activated RAB31 binds to the SPFH domain of FLOTs and drives the formation of ILVs through the flotillin domain, while the RAB31-FLOTs and Syntenin-Alix-ESCRT-III are two parallel exosome formation pathways, responsible for different cargoes, respectively (Kenific et al. 2021; Wei et al. 2021). In oligodendrocytes secreting exosome-related proteins, ESCRT function is not required for biogenesis and secretion of exosomes but rather depends on a ceramide-producing enzyme (Matsui et al. 2021; Tschuschke et al. 2020). In another cell system, tetraspanin CD63 is directly involved in ESCRT-independent sorting of melanocytes, rather than the traditional ESCRT-dependent cargo to form ILVs (van Niel et al. 2011). In conclusion, there are not only ESCRT-dependent but also ESCRT-independent mechanisms in the biogenesis of exosomes, which need to be further studied.

## Application of exosomes in spinal cord injury

Recently, exosomes derived from different cells have been extensively studied in SCI. Exosomes are involved in intercellular communication by carrying informational substances from the external environment or cells. Exosomes can be extracted by different methods such as differential ultracentrifugation, density gradient centrifugation, size exclusion chromatography, ultrafiltration, polyglycerol, and immunocapture (Varderidou-Minasian and Lorenowicz 2020). It can further be phenotyped by electron microscopy, nanoparticle tracking, flow cytometry, or western blotting. The extracted exosomes are injected into animals with SCI through tail vein, lumbar cistern, intrathecal, and scaffold to achieve the purpose of treatment (Herbert et al. 2022).

This function constitutes a novel pathway for the exchange of microenvironmental substances after SCI. Thus, exosomes can transport their cargo in recipient cells by activating various signaling pathways including within various mesenchymal stem cells (MSCs) and other related cells. First, MSCs-exosomes have anti-inflammatory effects, inhibit neuronal apoptosis, promote axonal regeneration, improve angiogenesis, and enhance macrophage/microglia polarization in the model of SCI (Kim et al. 2021). It has been observed that miR-126-modified bone marrow MSCs-exosomes may promote neurogenesis and reduce apoptosis, and promote angiogenesis and migration in human umbilical vein endothelial cells by impeding the appearance of SPRED1 and PIK3R2 (Huang et al. 2020). Wang et al. found that exosomes derived from human umbilical cord MSCs reduced the lesion area and enhanced neuroprotection through the miR-199a-3p/Cblb/TrkA and miR-145-5p/Cblb/TrkA pathways, respectively (Wang et al. 2021b). In addition, Lv’s research team showed that LncGm37494 is highly expressed in adipose tissue MSCs-exosomes under hypoxia, and its upregulation promotes the transition of microglia from M1 to M2 polarization by preventing miR-130b-3p and enhancing PPARγ expression (Shao et al. 2020). This suggests that adipose tissue MSCs-exosomes in the hypoxic state may promote functional recovery by suppressing inflammatory signaling. It has also been reported that MSCs-exosomes are used as drug delivery vehicles or combined with 3D hydrogels to enhance neural regeneration and attenuate glial scarring to achieve the purpose of repairing SCI (Cheng et al. 2021a; Zhang et al. 2021e). The application of MSCs-exosomes in the treatment of SCI has been sought after by many scholars, most of which are concentrated in animal experiments, and the safety of clinical application needs further research.

In addition to the powerful functions of MSCs-exosomes, exosomes derived from neural stem cells (NSCs), macrophages, astrocytes, and Schwann cells also play a significant role in SCI (Liu et al. 2021a). Chen et al. found that NSCs-exosomes loaded with FTY720 alleviated pathological variations and improved hindlimb function and hypoxia in model mice with SCI, and it also improved neuronal morphology, reduced inflammatory response and edema, and inhibited apoptosis (Chen et al. 2021a). Furthermore, Zhang et al. found that nerve growth factor is coupled to exosomes prepared from M2 macrophages through matrix metalloproteinase 9 and that carrying curcumin has anti-inflammatory and neuroprotective effects in secondary injury (Zhang et al. 2021c). In addition, activated astrocytes after SCI release CCL2, which acts on microglia and neurons through the exosome pathway, and then binds to CCR2 to enhance neuronal apoptosis and microglia activation (Rong et al. 2021b). Moreover, Schwann cell-exosomes may induce astrocytes to express TLR2 through NF-kB/PI3K signaling pathway, thereby reducing chondroitin sulfate proteoglycans deposition, and further promoting functional recovery (Pan et al. 2021). Additionally, exosomes derived from cerebrospinal fluid and plasma also have the functions of reducing apoptosis and regulating inflammatory responses (Khan et al. 2021; Kong et al. 2018). We summarize the relevant research on the application of exosomes in SCI from 2018 to 2022 for reference (Table 1).

**Table 1:** Application of exosomes in spinal cord injury

Source of exosomes	Exosome cargos	Targeted molecules/pathways	Related effect	References
BMSCs	miR-21-5p	FasL	Attenuated apoptosis and improved motor function	Zhou et al. (2019)
BMSCs	USP29	NRF2	Promoted M2-like microglia/macrophage polarization and regulated mitochondrial function	Liu et al. (2021b)
BMSCs	miR-124-3p	Ern1	Enhanced M2 polarization and impeded cell apoptosis	Li et al. (2020b)
BMSCs	miR-216a-5p	TLR4/NF-κB/PI3K/AKT	Shifted microglial polarization from M1 to M2 phenotype	Liu et al. (2020b)
BMSCs	miR-544	–	Abated histologic deficits and neuronal loss by attenuating inflammation	Li et al. (2020a)
BMSCs	miR-126	SPRED1, PIK3R2	Promoted neurogenesis and angiogenesis, reduced cell apoptosis, reduced lesion volume, and improved functional recovery	Huang et al. (2020)
BMSCs	Sonic hedgehog	–	Promoted neuronal regeneration	Jia et al. (2021b)
BMSCs	miR-181c	PTEN or NF-κB	Suppressed inflammation and apoptosis in microglia	Zhang et al. (2021f)
BMSCs	GIT1	PI3K/AKT	Restrained glial scar formation and neuroinflammation, attenuated apoptosis, and promoted axonal regeneration	Luo et al. (2021)
BMSCs	miR-338-5p	Cnr1/Rap1/Akt	Attenuated cell apoptosis and promoted neuronal survival	Zhang et al. (2021a)
BMSCs	miR-23b	TLR4/NF-κB	Inhibited inflammation response and improved hindlimb motor function	Nie and Jiang (2021)
BMSCs	miR-381	BRD4/WNT5A	Rescued neuron apoptosis and promoted functional recovery	Jia et al. (2021a)
BMSCs	siRNA	CTGF	Quenched inflammation and thwarted neuronal apoptosis and reactive astrocytes and glial scar formation	Huang et al. (2021a)
BMSCs	miR-26a	PTEN/Akt/mTOR	Improved neurogenesis and attenuated glial scarring	Chen et al. (2021b)
hucMSCs	miR-199a-3p, miR-145-5p	Cblb/NGF/TrkA, Cbl/NGF/TrkA	Decreased lesion size and emphasized the neuroprotective effect	Wang et al. (2021b)
hucMSCs	Paclitaxel	–	Enhanced neural regeneration and reduced scar deposition	Zhang et al. (2021e)
hucMSCs	miR-29b-3p	PTEN/Akt/mTOR	Reduced pathological changes, improved motor function, and promoted nerve function repair	Xiao et al. (2021)
hMSCs, PC12 cells	miR-21 and miR-19b	PTEN	Inhibited apoptosis of neuronal cells by downregulating PTEN expression	Xu et al. (2019)
hucMSCs	CD73	cAMP/PKA	Promoted microglia cell polarization from M1 to M2 phenotype, reduced activation of astrocytes	Zhai et al. (2021)
MSCs	miR-21	PTEN/PDCD4	Suppressed neuron cell death and improved functional recovery	Kang et al. (2019)
ADMSCs	miR-133b	–	Enhanced axon regeneration and promoted recovery of neurological function	Ren et al. (2019b)
ADMSCs	LncGm37494	miR-130b-3p/PPARγ	Suppressed inflammatory factor expression, promoted functional recovery, and shifted microglia from M1 to M2 polarization	Shao et al. (2020)
Macrophages	NADPH oxidase 2 complexes	NOX2/PTEN/PI3K/p-Akt	Promoted axonal outgrowth and regeneration of sensory neurons	Hervera et al. (2018)
Macrophages	Berberine (drug)	–	Reduced inflammatory and apoptotic cytokines	Gao et al. (2021)
Macrophages	NGF and curcumin	–	Anti-inflammatory and neuroprotective properties	Zhang et al. (2021c)
NSCs	IGF-1	miR-219a-2-3p/YY1	Inhibited neuroinflammation and promoted neuroprotective effect	Ma et al. (2019)
NSCs	VEGF-A	–	Accelerated microvascular regeneration and reduced spinal cord cavity	Zhong et al. (2020a)
NSCs	FTY720	PTEN/AKT	Ameliorated morphology of neurons, reduced inflammatory infiltration and edema, and inhibited cell apoptosis	Chen et al. (2021a)
Neuron	miR-124-3p	MYH9/PI3K/AKT/NF-κB	Suppressed activation of M1 microglia and A1 astrocytes	Jiang et al. (2020)
NG2 + cells	Retinoic acid	EGFR-calcium	Promoted remyelination and oligodendrocyte differentiation	Goncalves et al. (2019)
Astrocytes	CCL2	CCR2	Aggravated microglia activation and neuronal apoptosis	Rong et al. (2021b)
Human urine stem cell	ANGPTL3	PI3K/AKT	Enhanced neurological functional recovery and promoted angiogenesis	Cao et al. (2021)

## Overview of autophagy

Autophagy is a ubiquitous process in almost all eukaryotes. Under normal physiological conditions, autophagy degrades intracellular proteins and damaged organelles to sustain the stability of intracellular environment and the survival of cells. In the case of cells facing starvation, hypoxia, or lack of growth factors, autophagy is induced to recover amino acids and fatty acids to uphold cell survival (Zheng 2017). Autophagy is also involved in the pathological process of many diseases, such as tumors, diabetes, and neurological diseases (Fîlfan et al. 2017; Medvedev et al. 2017; Shrivastava et al. 2016). When the pathological state changes and the cell environment changes, cell autophagy also changes accordingly. Autophagy can be used not only as a “modulator” in the pathophysiological process but also as a “target” for other effects. Moderate autophagy is essential for cell homeostasis, but excessive autophagy leads to cell death and accelerates disease progression (Zhou et al. 2020a). This indicates that autophagy has both protective and harmful functions in physiological and pathological environments.

Genes required for autophagy, called autophagy associated genes (ATG), were first discovered in yeast (Tsukada and Ohsumi 1993). Today, in the evolution of eukaryotes, more than 30 highly conserved homologous ATG have been identified, and they are involved in a series of autophagy processes (Glick et al. 2010). Autophagy is induced by nutrient deficiency, lack of growth factors, or reactive oxygen species (ROS). At the same time, an assortment of signal pathways is involved in regulating the occurrence of autophagy. Nutrients are the most common autophagy modulator and are regulated by the rapamycin (mTOR inhibitor) targeting (Chen et al. 2020b). When nutrients and growth factors are abundant, mTOR complex 1 (mTORC1) hinders autophagy by phosphorylation and ULK1 (unc-51 like autophagy activating kinase 1) (Park et al. 2016). After autophagy is induced, the ATG1 and ULK1 complexes initiate early autophagosome membrane assembly and recruit ATG6 (Beclin1 in mammals), where ATG6 synthesizes phosphatidylinositol 3-phosphate (PI3P) to induce the expansion of phagocytes (Matsuura et al. 1997). In addition, two ubiquitin complexes composed of ATG5-ATG12 and ATG7-ATG3 complexes covalently link ATG8 (microtubule associated protein 1 light chain 3β, LC3β) with phosphatidylethanolamine on the autophagosome membrane (Ichimura et al. 2000). Subsequently, the formed autophagosomes fuse with lysosomes to finally form autolysosomes, in which the components are hydrolyzed by acid hydrolase so that the substances can be recycled.

Besides, its degradation function, autophagy is also involved in the pathway of cytoplasmic protein secretion. This method is different from the conventional secretion pathway from ER to the Golgi apparatus but requires the participation of the plasma membrane of signal peptide sequence. For example, the precursor of the pro-inflammatory factor interleukin-1β (IL-1β) lacks an amino acid signal sequence in the cytoplasm and is activated by the inflammasome (Schroder and Tschopp 2010). When IL-1β is secreted, it interacts with autophagosomes, is recognized by the specific receptor TRIM16 that interacts with R-SNARE Sec22b, and is then transported to the LC3-II + membrane, and finally fused with the plasma membrane to be released (Kimura et al. 2017). Among them, MVBs are essential for autophagy-dependent IL-1β secretion. In general, autophagy plays an imperative role in regulating the secretion of conventional and unconventional substances and plays an important role in performing cell functions and participating in communication between cells.

## Application of autophagy in spinal cord injury

Autophagy has been described to promote cell death in SCI models, but it has also been described that autophagy has a cytoprotective effect and can reduce cell death (Table 2). With the comprehensive study of autophagy, a detailed understanding of the function of autophagy after SCI (Ray 2020). Rapamycin was found to promote the expression of Beclin1 and LC3β and reduce apoptosis, suggesting enhanced autophagy and motor function recovery (Liu et al. 2020a). In the rat SCI model, it was also found that rapamycin enhanced autophagy by preventing mTOR signaling, reduced nerve tissue injury, and promoted the recovery of nerve function (Chen et al. 2013). In contrast, autophagy-induced cell death is thought to be associated with neural tissue damage (Kanno et al. 2012). Kanno et al. found that the autophagosome membrane marker protein LC3β was significantly increased in neuronal cells, astrocytes, and oligodendrocytes (Kanno et al. 2011). It is speculated that autophagy of nerve cells leads to the reduction of nerve cells. Walker et al. found that the PTEN lipid phosphatase inhibitor bpV (pic) activates Akt/mTOR signaling pathway to inhibit autophagy and improves recovery of hindlimb motor function (Walker et al. 2012).

**Table 2:** Application of autophagy in spinal cord injury (↑:activation, ↓:inhibition)

Cell or animal model	Gene or drugs	Targeted molecules/pathways	Autophagy	Related effect	References
PC12, neurons	Fosl1	AMPK	↑	Improved neurological function and inhibited inflammation and inflammation	Zhong et al. (2022)
PC12, neurons	sestrin2	AMPK/mTOR	↑	Limited endoplasmic reticulum stress and promoted neuronal survival	Li et al. (2021d)
Neurons	USP11	Beclin1	↑	Promoted ferroptosis	Rong et al. (2021a)
Neurons	Ozone	NRF2/ARE	↑	Protected spinal cord neuron from injury	Zhang et al. (2021d)
Mouse	Triptolide	MAPK/ERK1/2	↑	Reduced neuronal cell death	Zhu et al. (2020a)
Mouse	Betulinic acid	AMPK/mTOR/TFEB	↑	Eliminated the accumulation of ROS and inhibited pyroptosis	Wu et al. (2021)
Mouse	GDF-11	TFE3	↑	Stimulated autophagy improvement and inhibited pyroptosis and necroptosis	Xu et al. (2021b)
Mouse, neurons, PC12	Brd4	AMPK/mTOR/ULK1	↑	Reduced oxidative stress and inhibited the expression of apoptotic proteins to promote neural survival	Li et al. (2020c)
Mouse, PC12	TFE3	AMPK/mTOR and AMPK/SKP2/CARM1	↑	Ameliorated ER stress-induced apoptosis in neurons	Zhou et al. (2020a)
Mouse, PC12	HDAC6	–	↑	Increased axonal length	Zheng et al. (2020)
Mouse, oligodendrocytes	MICAL1	Nrf2	↑	Protected oligodendrocytes from apoptosis	Xu et al. (2021a)
Mouse, VSC4.1	Amlodipine	–	↑	Alleviated apoptosis and neuronal loss	Huang et al. (2021b)
VSC4.1	Resveratrol	SIRT1	↑	Inhibited motoneuronal apoptosis	Tian et al. (2021)
Rat	IF	AMPK/mTOR	↑	Exerted a neuroprotective effect and enhanced lysosome function	Yuan et al. (2021)
Rat	Trehalose	mTOR	↑	Inhibited apoptosis, reduced lesion cavity expansion, decreased neuron loss	Zhou et al. (2021b)
Rat	Quercetin	Akt/mTOR/p70S6k	↑	Promoted locomotor function recovery, axonal regeneration, and energy metabolism	Wang et al. (2021c)
Rat	Curcumin	Akt/mTOR	↑	Reduced neuron apoptosis, improved remyelination, and suppressed the inflammatory response	Li et al. (2021a)
Rat	EPO	ERK	↑	Reduced cavity ratio, cell apoptosis, and motor neuron loss	Zhong et al. (2020b)
Rat	Ezetimibe	PI3K/AKT/mTOR	↑	Improved functional recovery, neuronal survival, and morphological characteristics	Chen et al. (2020a)
Rat	Liraglutide	AMPK/FOXO3	↑	Enhanced motor function recovery and alleviated the degree of necrosis and loss of motor neurons	Zhang et al. (2020)
Rat	Scopoletin	AMPK/mTOR	↑	Alleviated neuronal apoptosis	Zhou et al. (2020b)
Rat	Melatonin	SIRT1/AMPK	↑	Exerted neuroprotective effect	Gao et al. (2020a)
Rat, SH-SY5Y	Pyr-PDS	PDH2/HIF-1α/BNIP3	↑	Protective effects	Xiong et al. (2020)
Rat, SY-SH-5Y	LncRNA TCTN2	miR-216b/Beclin-1	↑	Reduced neuron apoptosis	Ren et al. (2019a)
Rat, HAPI, PC12	AS-IV	mTORC1	↑	Promoted M2 microglial polarization to attenuate inflammatory response and reduced neuronal apoptosis	Lin et al. (2020)
Rat, neurons	Wnt-3a	mTOR	↑	Reduced the loss of spinal anterior horn motor neurons	Gao et al. (2020b)
Rat, neurons	Melatonin	PI3K/AKT/mTOR	↑	Reduced apoptosis and improved motor function recovery	Li et al. (2019)
Rat, RNSC	bpV	ERK1/2	↑	Decreased neuronal apoptosis	Tang et al. (2019)
MECs	GIT1	VEGF	↑	Enhanced myelin debris clearance and angiogenesis	Wan et al. (2021)
Rat	NT-3	–	↓	Promoted oligodendrocytes proliferation	Cong et al. (2020)
Rat	Apelin-13	–	↓	Suppressed mitochondrial dysfunction, resisted oxidative stress	Xu and Li (2020)
Rat	FGF21	–	↓	Attenuated cell death and promoted the functional recovery	Zhu et al. (2020b)
Rat, neurons	Ang-1	–	↓	Neuroprotective role	Yin et al. (2019)
Rat, PC12	miR-384-5p	Beclin-1	↓	Increased spinal cord neuron survival and inhibited endoplasmic reticulum stress	Zhou et al. (2020c)
PC12	SNHG1	miR-362-3p/Jak2/stat3	↓	Regulated cell viability and suppressed apoptosis	Zhou et al. (2021a)
Mouse	PARP	pI3K/pAkt	↓	Slowed cell death and decreased autophagy-activation proteins	Casili et al. (2020)
Mouse, HUVECs	PDGF	PDGFR/AKT	↓	Reduced neuronal apoptosis and astrocyte proliferation, increased collagen synthesis	Ye et al. (2021)
NSCs	bFGF	–	↓	Promoted neuronal regeneration and inhibited glial scar formation	Zhu et al. (2021)
Astrocytes	TGF-β	–	↓	Reduced CSPG secretion and improved axonal regeneration	Alizadeh et al. (2021)

Autophagy plays different roles in regulating various glial cells following SCI. Oligodendrocytes are a type of glial cells, mainly derived from the oligodendrocyte precursor cells. The death and demyelination by oligodendrocytes are critical points in the pathological process of secondary injury (Lipinski et al. 2015). To investigate the role of autophagy after SCI, it was found that decreased oligodendrocyte autophagy flux and restoration of hindlimb motor function in ATG5 knockout transgenic mice (Saraswat Ohri et al. 2018). Therefore, autophagy is an essential cytoprotective pathway in oligodendrocytes. Studies have shown that loss of ATG5 severely impairs the survival of oligodendrocyte precursor cells and inhibits their differentiation into oligodendrocytes. Mature oligodendrocytes express high levels of autophagy-related markers, indicating that the occurrence of autophagy is inextricably linked to the formation of oligodendrocyte myelin (Bankston et al. 2019). The results suggest that autophagy is indispensable for oligodendrocyte differentiation and survival and normal myelination.

Inflammatory responses in secondary injury after SCI are mainly mediated by activated microglia/macrophages. The microglia/macrophage phenotype is divided into M1 phenotype and M2 phenotype (Guo et al. 2019). Following SCI, microglia polarize predominantly to the M1 phenotype, resulting in the release of neurotoxic substances such as free radicals, inflammatory cytokines, and chemokines. These inflammatory mediators aggravate the inflammatory response and further accelerate neuronal cell death (Wang et al. 2018). Conversely, the M2 phenotype suppressed immune responses and promoted nerve regeneration by releasing IL-4 and TGF-β (Gaudet and Fonken 2018; Orr and Gensel 2018). Therefore, the different mechanisms of microglia/macrophages in the inflammatory response after SCI deserve further exploration. Studies have shown that autophagy mediated by the AMPK/mTOR signaling pathway activates the polarization of microglia/macrophages from M1 to M2 to protect neuronal cells from apoptosis (Wang et al. 2018). Activated autophagy reduces the number of activated microglia, thereby inhibiting the excretion of proinflammatory cytokines TNF-α, IL-1β, and IL-6 to reduce neuroinflammatory responses (Rong et al. 2019b). Autophagy is also thought to modulate the activity of the NLRP3 inflammasome and decrease IL-1β-induced cellular responses (Cho et al. 2014). Furthermore, autophagy limits mitochondrial damage and ROS production. For example, autophagy inhibition may be involved in the hyperactivation of microglia or macrophages, and the production of mitochondrial ROS may be involved in regulating the pro-inflammatory response of microglia (Ye et al. 2017). Conversely, the inflammatory microenvironment may also regulate autophagy levels. For example, in the lipopolysaccharide-induced microglia inflammation model, the expression of autophagy-related proteins LC3β and p62 is increased.

After SCI, primitive astrocytes sequentially exhibited distinct phenotypes, first reactive astrocytes, and then scar-forming astrocytes. Little is known about the mechanism or function of astrocyte autophagy. Related research hints at a possible link between astrocytes and autophagy (Alizadeh et al. 2021). However, the death of reactive astrocytes in secondary injury may be through a process of necroptosis rather than autophagy (Fan et al. 2016). It is necessary to further study how autophagy affects the survival and function of astrocytes to promote functional recovery after SCI.

## Crosstalk between exosomes and autophagy in spinal cord injury

Studies have shown that there are shared molecules or organelles between autophagy and exosome biogenesis (Fig. 1), which have important implications for physiological and pathological processes (Tooze et al. 2014). Furthermore, the reciprocal communication between autophagy and exosome biogenesis is principally dependent on the body’s internal environment, and they work together in removing unwanted proteins and can complement each other’s deficiencies. In recent years, some studies have pointed out that autophagy-related molecules are involved in the biogenesis of exosomes. For instance, ATG5 and ATG16L1 play important roles in exosome biogenesis. ATG5 mediates the dissociation of the proton pump (V_1_V_0_-ATPase) from MVBs, thereby avoiding the acidification of MVBs lumen (Guo et al. 2017). This is because the regulatory component ATP6V1E1 is detached from V_1_V_0_-ATPase and enters exosomes to promote the fusion of MVBs with the plasma membrane and then enhance the excretion of exosomes. Indeed, knockdown of ATG5 and ATG16L1 inhibited the discharge of exosomes and greatly reduced the content of lipidated LC3β in exosomes. Furthermore, treatment with lysosome or V-ATPase inhibitors saved exosome secretion in ATG5 knockout cells, whereas this was not found in ATG7 knockout cells (Alvarez-Erviti et al. 2011; Guo et al. 2018). This implicates that the potential function of autophagy-related proteins is to inhibit their degradation in lysosomes by controlling the acidification of MVBs (Martinez et al. 2015). Meanwhile, exosomes produced through autophagy-dependent pathways have special selectivity for autophagy-related genes.

It is worth mentioning that the ATG12-ATG3 complex, which catalyzes LC3β binding, adjusts exosome biogenesis by interacting with ALIX (Murrow et al. 2015). ALIX, an ESCRT-related protein, plays a crucial role in the process of exosome transport and signaling (Baietti et al. 2012). Studies indicate that ATG12-ATG3 complex deficiency may alter the morphology of MVBs, hinder late endosomal trafficking, and thus reduce exosome biogenesis. Furthermore, ATG12-ATG3 complex deficiency reduced basal autophagic flux, suggesting a reciprocal regulatory relationship between autophagy and exosome biogenesis. Interestingly, disassembly of ALIX or the ATG12-ATG3 complex did not affect starvation-induced autophagy levels. This suggests that physiological and stress-induced autophagy are regulated by different mechanisms, and the mechanisms of crosstalk between autophagy and exosomes for different forms of cellular stress response remain unknown. In addition, Bader et al. demonstrated that ATG9, the only transmembrane ATG, is required for the formation of ILVs in *Drosophila* (Bader et al. 2015). Deletion of ATG9 inhibits autophagic flux and reduces the content of ILVs in amphisome and autolysosomes. Another complex, class III PI3K, has shared mechanisms in exosome biogenesis and autophagy pathways, as Beclin-1 knockdown prevented exosome secretion and inhibited autophagy in chronic myeloid leukemia cells (Liu et al. 2016). Similarly, SNARE proteins that mediate autophagosome generation enhances the fusion of MVBs to the plasma membrane, supporting crosstalk between autophagy and exosomes, but more evidence is needed (Adnan et al. 2019). Different molecules involved in the crosstalk between autophagy and exosomes are gradually being discovered and studied, and whether this crosstalk mechanism exists in different diseases needs to be further elucidated.

The amphisome formed by the synthesis of autophagosomes with MVBs appears to serve as a transport hub for autophagy and exosome biogenesis, which eventually bind to lysosomes for degradation. Studies have found that there is an antagonistic relationship between autophagy and exosome secretion. In the leukemia cell line K562, autophagy induced by starvation or rapamycin increased autophagosomes fusion to MVBs, thereby reducing exosome release (Fader et al. 2008). Notably, inhibiting the excretion of exosomes promoted the transport of MVBs towards the autophagy pathway. For example, the ubiquitin-like protein ISG15 induces fusion of MVBs with lysosomes to promote protein aggregation and degradation, and then ISG15 inhibits exosome secretion by mediating TSG101, an ESCRT accessory protein (Villarroya-Beltri et al. 2016). Further, bafilomycin A1 inhibited the interaction between MVBs and lysosomes to prevent autophagy, thereby restoring exosomes release in ISGylation-treated cells. This suggests that autophagy is involved in the lysosome-based degradation of MVBs, but does not affect the biogenesis of MVBs. Furthermore, Hurwitz et al. demonstrated that CD63 knockout promoted autophagic clearance of abnormal endocytic vacuoles in cells, whereas inhibition of autophagy restored exosome biogenesis (Hurwitz et al. 2018). These findings suggest that exosome biogenesis and autophagy pathways share molecular mechanisms that require further study.

It was found that vesicles are secreted into the extracellular space by fusion of the amphisome with the plasma membrane, rather than the fusion of MVBs with the plasma membrane. For example, IFN-γ-induced autophagy is critical for lung epithelial cells to express annexin A2 (ANXA2) produced through an unconventional exosome release pathway. ANXA2 is captured by autophagosomes with the participation of autophagy-related proteins LC3β and ATG5. Following, RAB11 stimulated the fusion of ANXA2-loaded autophagosomes with MVBs to form ANXA2-loaded amphisome, which further fused with the plasma membrane with the participation of RAB8A and RAB27A to release ANXA2-loaded exosomes (Chen et al. 2017). However, we must distinguish autophagy-dependent unconventional secretion from exosomal release. Although functional MVBs are essential for autophagy-dependent IL-1β discharge, autophagosome-lysosome fusion is not necessarily present during this process (Florey et al. 2011). This implies that LC3β-positive IL-1β carrier vesicles may directly fuse with the plasma membrane, whereas the dependence on MVBs function may be due to the reciprocal communication between autophagy and endocytosis. Interestingly, RAB8A not only regulates autophagy-dependent IL-1β vesicles secretion but also participates in IFN-γ-induced ANXA2 exosome biogenesis (Dupont et al. 2011; Kowal et al. 2014). These results suggest that there may be an intersection between the autophagy-mediated unconventional secretion pathway and the exosome secretion pathway, in which many unknown molecules remain.

Numerous studies have suggested that the link between exosomes and autophagy affects the occurrence and development of diseases. For example, BMSCs-EVs inhibited the autophagy of cardiomyocytes induced by ischemia and hypoxia, exosomes carrying miRNA-122a alleviated renal fibrosis by inhibiting autophagy (Li et al. 2022), and autophagy-related GPR64-enriched exosomes stimulated NF-κB signaling to enhance cancer cell invasiveness (Xi et al. 2021). There are few studies on the crosstalk between exosomes and autophagy in SCI (Table 3). Exosome-delivered cargo exerts anti-apoptotic, anti-inflammatory, and axon-protective roles in SCI by regulating autophagy. Rong et al. demonstrated that neural stem cell-derived exosomes carrying 14–3-3t protein (NSC-14–3-3t-exo) improved cellular anti-apoptotic and anti-inflammatory effects in SCI, and found that autophagy-related protein LC3IIβ increased and P62 decreased (Rong et al. 2019a, b). Further, the number of autophagosomes was significantly increased in neuronal cells treated with NSC-14–3-3t-Exos, and autophagy was activated by interacting with Beclin-1. In addition, a substantial increase in the number of autophagosomes was observed in neuronal cells treated with exosomes derived from bone marrow mesenchymal stem cells (BMSCs-Exos), while stimulating the expression of autophagy-related proteins LC3IIβ and Beclin-1, alleviating neuronal apoptosis following SCI (Gu et al. 2020). On the other hand, BMSCs-Exos carrying miR-455-5p activated autophagy by targeting Nogo-A, reduced neuronal apoptosis, and improved the recovery of hindlimb function in rats with SCI (Liu et al. 2022). Similarly, exosomes extracted from bone marrow-derived M2 macrophages increased the expression of LC3IIβ and inhibited neuronal apoptosis by carrying miR-421-3p and binding to the mTOR pathway (Wang et al. 2020). Concomitantly, tail vein injection of inhibitors carrying miR-421-3p exosomes reduced neuronal autophagic flux in SCI mice. Zhang et al. found that peripheral macrophage-derived exosomes stimulated the expression of LC3II/I and Beclin-1 in microglia by constraining the PI3K/AKT/mTOR signaling pathway, and promoted M2-type microglia polarization (Zhang et al. 2021b). This implies that peripheral macrophage-derived exosomes attenuate the expression of inflammatory factors by inducing autophagy after SCI. Interestingly, primary microglia-derived exosomes can efficiently carry resveratrol across the blood–brain barrier to assist in the recovery of paralyzed hindlimbs in rats, which may be related to the activation of autophagy and inhibition of apoptosis by regulating the PI3K pathway (Fan et al. 2020). Alternatively, Schwann cell-derived exosomes promoted axonal regeneration by inducing autophagy and reducing apoptosis after SCI, which may be related to the EGFR/Akt/mTOR pathway (Pan et al. 2022). Although these studies suggest the relevance of autophagy and exosome biogenesis in the treatment of SCI, the molecular mechanism of crosstalk remains unclear and needs to be further explored in the future.

**Table 3:** Crosstalk between exosomes and autophagy in spinal cord injury

Source of exosomes	Exosome cargos	Targeted molecules/pathways	Autophagy	Related effect	References
NSCs	14–3-3t	Beclin-1	Activation	Enhanced anti-apoptotic and anti-inflammatory	Rong et al. (2019a)
BMSCs	miR-455-5p	Nogo-A	Activation	Improved the recovery of hindlimb function and reduced apoptosis of neurons	Liu et al. (2022)
BMSCs	–	–	Activation	Attenuated neuronal apoptosis	Gu et al. (2020)
Primary microglia	Resveratrol	PI3K	Activation	Inhibited apoptosis of neurons and assisted rehabilitation of paralyzed limbs in rats	Fan et al. (2020)
Schwann	–	EGFR/Akt/mTOR	Activation	Promoted axonal protection, decreased apoptosis	Pan et al. (2022)
M2-BMDM	miR-421-3p	mTOR	Activation	Reduced neuronal apoptosis and attenuated tissue damage	Wang et al. (2020)
Peripheral macrophages	–	PI3K/AKT/mTOR	Activation	Promoted anti-inflammatory type microglial polarization	Zhang et al. (2021b)

## Conclusions and future directions

Both exosomes and autophagy pathways play anti-apoptotic, anti-inflammatory, and axonal protection roles after SCI. The exosome biogenesis pathway is related to autophagy in different ways, including the synthesis of amphisome with lysosomes to degrade carried cargo, where autophagy-related proteins may be involved in the secretion of exosomes. Autophagy and exosome secretion are in stable equilibrium under normal physiological conditions. However, exosome formation and release are enhanced in the presence of impaired autophagy as an alternative approach to reduce cellular stress and maintain cellular homeostasis. Conversely, activation of autophagy inhibited the release of exosomes. Since exosome formation and autophagy have overlapping pathways, activation of autophagy diverts more MVBs into the autophagy pathway, thereby reducing exosome secretion. In addition, autophagy may disrupt the material structure between cells, leading to cell death, while exosomes carrying specific components can repair cell damage and promote cell proliferation.

The interconnection between exosomes and autophagy has achieved some results in the treatment of some diseases, but there is still no clear answer to the safety and dosage of exosome implantation. Based on the connection between autophagy and exosomes in SCI, it is speculated that there is a molecular sharing mechanism between them. The pathological process of SCI involves inflammation, axonal loss, neuronal necrosis and apoptosis, and disruption of vascular endothelial integrity. From this, we wondered whether it would be possible to engineer exosomes (such as those containing autophagy-related miRNAs) to regulate autophagy in various cells, or to use exosomes with specific components to repair autophagy-induced damage after SCI. Here, we briefly summarize the applications between autophagy and exosome biogenesis in recent years and propose new possible ideas for the early treatment of SCI.

## Abbreviations

SCI: Spinal cord injury
EVs: Extracellular vesicles
ILVs: Intraluminal vesicles
MVBs: Multivesicular bodies
LEs: Late endosomes
ESCRT: Endosomal sorting complex required for transport
Vps4: Vacuolar protein sorting-associated protein 4
Tsg101: Tumor susceptibility 101
Alix: ALG-2 interacting protein X
MSCs: mesenchymal stem cells
NSCs: neural stem cells
hMSCs: human mesenchymal stem cells
hucMSCs: human umbilical cord mesenchymal stem cells
BMSCs: bone marrow mesenchymal stem cells
ADMSCs: adipose-derived mesenchymal stem cell-derived exosomes
EFMSCs: epidural fat mesenchymal stem cells
hWJMSCs: human Wharton’s jelly mesenchymal stem cells
hPMSCs: human placenta mesenchymal stem cells
MFMSCs: macrophage membrane-fused umbilical cord blood-derived MSCs
ATG: autophagy associated genes
ROS: reactive oxygen species
LC3β: microtubule associated protein 1 light chain 3β
IL-1β: interleukin-1β
PTEN: phosphatase and tensin homologs
TGF-β: transforming growth factor-β
ANXA2: annexin A2
IFN-γ: interferon-γ
TNF-α: tumor necrosis factor α
